# Analysis of serological data to investigate heterogeneity of malaria transmission: a community-based cross-sectional study in an area conducting elimination in Indonesia

**DOI:** 10.1186/s12936-019-2866-z

**Published:** 2019-07-08

**Authors:** Henry Surendra, Mahardika A. Wijayanti, Elsa H. Murhandarwati, Irnawati, Titik Yuniarti, Mardiati, Herdiana, Maria E. Sumiwi, William A. Hawley, Neil F. Lobo, Jackie Cook, Chris Drakeley, Supargiyono

**Affiliations:** 1 Infection Biology Department, London School of Hygiene and Tropical Medicine, London, WC1E 7HT UK; 2 Centre for Tropical Medicine, Faculty of Medicine, Public Health and Nursing, Universitas Gadjah Mada, Yogyakarta, 55281 Indonesia; 3 Department of Parasitology, Faculty of Medicine, Public Health and Nursing, Universitas Gadjah Mada, Yogyakarta, 55281 Indonesia; 4 Sabang Municipal Health Office, Sabang, Aceh Indonesia; 5 Child Survival and Development Cluster, UNICEF Aceh Field Office, Jakarta, Indonesia; 6 Child Survival and Development Cluster, UNICEF Indonesia Country Office, Jakarta, Indonesia; 7 Eck Institute for Global Health, University of Notre Dame, Notre Dame, IN USA; 8 MRC Tropical Epidemiology Group, Department of Infectious Disease Epidemiology, London School of Hygiene and Tropical Medicine, London, WC1E 7HT UK

**Keywords:** Serology, Epidemiology, Surveillance, Malaria, *P. falciparum*, *P. vivax*, Elimination

## Abstract

**Background:**

Analysis of anti-malarial antibody responses has the potential to improve characterization of the variation in exposure to infection in low transmission settings, where conventional measures, such as entomological estimates and parasitaemia point prevalence become less sensitive and expensive to measure. This study evaluates the use of sero-epidemiological analysis to investigate heterogeneity of transmission in area conducting elimination in Indonesia.

**Methods:**

Filter paper bloodspots and epidemiological data were collected through a community-based cross-sectional study conducted in two sub-districts in Sabang municipality, Aceh province, Indonesia in 2013. Antibody responses to merozoite surface protein 1 (MSP-1_19_) and apical membrane antigen 1 (AMA-1) for *Plasmodium falciparum* and *Plasmodium vivax* were measured using indirect enzyme-linked immunosorbent assay (ELISA). Seroconversion rates (SCR) were estimated by fitting a simple reversible catalytic model to seroprevalence data for each antibody. Spatial analysis was performed using a Normal model (SaTScan v.9.4.2) to identify the clustering of higher values of household antibody responses. Multiple logistic regression was used to investigate factors associated with exposure.

**Results:**

1624 samples were collected from 605 households. Seroprevalence to any *P. falciparum* antigen was higher than to any *P. vivax* antigen, 6.9% (95% CI 5.8–8.2) vs 2.0% (95% CI 1.4–2.8). SCR estimates suggest that there was a significant change in *P. falciparum* transmission with no exposure seen in children under 5 years old. *Plasmodium falciparum* SCR in over 5 years old was 0.008 (95% CI 0.003–0.017) and 0.012 (95% CI 0.005–0.030) in Sukakarya and Sukajaya sub-districts, respectively. Clusters of exposure were detected for both *P. falciparum* and *P. vivax,* most of them in Sukajaya sub-district. Higher age, *P. vivax* seropositivity and use of long-lasting insecticide-treated bed net (LLIN) were associated with higher *P. falciparum* exposure.

**Conclusion:**

Analysis of community-based serological data helps describe the level of transmission, heterogeneity and factors associated with malaria transmission in Sabang. This approach could be an important additional tool for malaria monitoring and surveillance in low transmission settings in Indonesia.

**Electronic supplementary material:**

The online version of this article (10.1186/s12936-019-2866-z) contains supplementary material, which is available to authorized users.

## Background

In recent years, there has been a decline in malaria transmission in many regions, leading to optimism that malaria elimination might be achieved in numerous countries [1–8]. As transmission declines, monitoring changes in malaria transmission intensity and disease prevalence through surveillance systems becomes increasingly important to allow the evaluation of health services and control programs [9, 10]. The latest World Health Organization (WHO) malaria surveillance manual confirms that improved surveillance is a major component of the WHO strategy [11]. However, conventional measures such as entomological estimates and parasitaemia point prevalence become less sensitive and relatively more expensive as transmission declines [12, 13]. Disease surveillance is further compounded by difficult access to remote and isolated communities, increased risks in forest workers and other highly mobile populations and the difficulties of tracking cross-border movements [14–20].

An additional approach to measure malaria transmission is to detect anti-malarial antibodies, which provide a marker for exposure to malaria [9]. Malaria infections generate antibodies which can be detected for several months and years after the infection has been resolved. Although serology is unlikely to be useful for diagnosing actively infected individuals because antibodies take days to develop and then persist after infection [9, 13], detection of these antibodies indicates previous exposure and offers an additional, more sensitive measure of infection and transmission, particularly in low endemic settings where the sensitivity of parasitological tools is inadequate [21–25] and gold standard tests like the parasite rate and the entomological inoculation rate (EIR), may have insufficient statistical power unless the sampling is intensively done [26–28]. This approach has been utilized in several countries and reported as a more sensitive tool to assess population-level malaria exposure in low-transmission settings [9, 13].

Seroconversion rate (the proportion of people in the population who are expected to seroconvert each year) is a serological parameter used to understand malaria transmission dynamics. Previous studies found that seroconversion rate (SCR) provides a proxy measure for estimating the transmission intensity in a community as it was strongly correlated with the EIR and annual parasite incidence collected by the malaria surveillance programme [10, 14]. Serological estimates of transmission have been utilized in many low endemic settings, including Indonesia [29, 30], and have additionally been used to identify populations at higher risk of malaria exposure [9, 31], foci of transmission [32, 33] and to describe historical changes in disease burden [25]. While there is great promise in this approach, it needs further refinement.

Recent studies have reported the potential use of recombinant Merozoite Surface Protein 1 (PfMSP-1_19_) and Apical Membrane Antigen 1 (PfAMA-1) as serological parameters to assess malaria transmission intensity in Indonesia. First, a population-based cross-sectional study conducted in three different endemicity areas showed the potential application of these methods for detecting changes in transmission exposure, particularly in lower transmission settings and with less immunogenic antigens (such as PfMSP-1_19_) [30]. Second, a cohort study of Indonesian schoolchildren found that it is possible to assess the interruption of transmission by measuring seroconversion rates from individual-level longitudinal data on antibody titres [29]. These studies suggested serological analysis has the potential to assess malaria burden and heterogeneity of infections in the Indonesian population. As antibodies to AMA-1 and MSP-1_19_ antigens have been reported to persist for several years after infection and in the absence of reinfection, any antibodies detected in younger children would reflect more recent infection in low transmission settings [10]. Therefore, as Indonesia aims to eliminate malaria by 2030, further implementation and evaluation of sero-epidemiological analysis in areas moving towards elimination would garner valuable information for malaria control programmes. This study explores the use of sero-epidemiological analysis for assessing the intensity and heterogeneity of malaria transmission as well as factors associated with malaria exposure in an area conducting elimination in Indonesia.

## Methods

### Study site

**Fig. 1:**
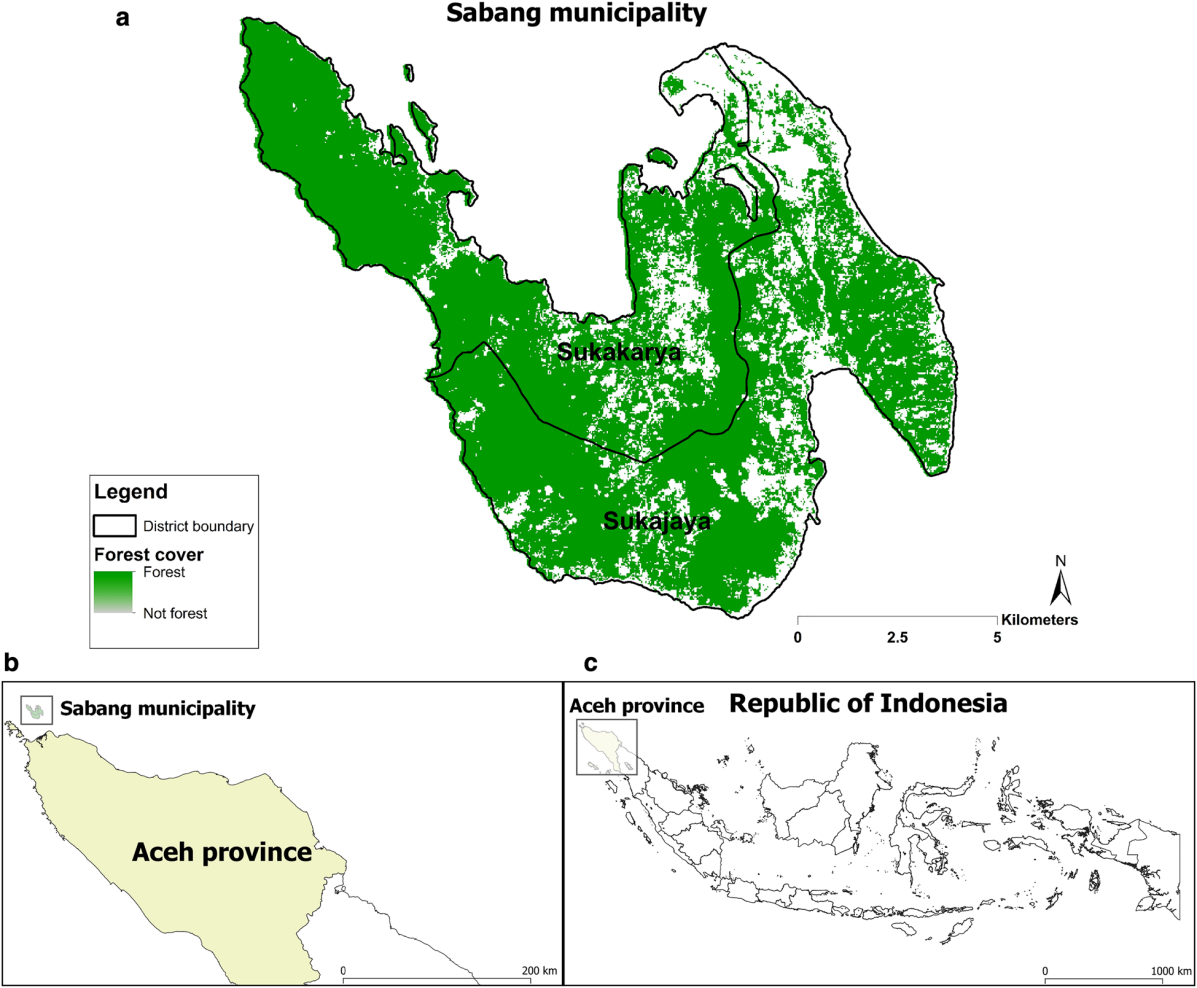
Map showing study sites in two sub-districts in Sabang municipality, Aceh province, Indonesia (**a**). Inset maps showing geographical location of Sabang municipality within Aceh province (**b**), and location of Aceh province within Indonesian archipelago (**c**)

The study was conducted in Sabang municipality, Aceh province, Indonesia (Fig. 1). The municipality is located at the north-westernmost part of Indonesia and is part of Aceh province. The municipality has an area of 153 km^2^ covering five islands but only the largest island, Weh, is permanently inhabited. The population on Weh island is approximately 30,000 and it is divided administratively into two sub-districts (Sukakarya and Sukajaya) with 18 villages. Sabang has a very low-level annual parasite incidence, 0.13 per thousand population in 2011. Based on its geographic position at the western end of the archipelago, its diverse mosquito fauna, the presence of both major malaria parasites, and its strong local government, Sabang municipality was considered as an appropriate place to pilot malaria elimination in Indonesia [34].

### Study design and data collection

Community-based cross-sectional surveys were performed during the malaria transmission season between October and December 2013. Households list were obtained from local authorities and were arbitrarily assigned numbers according to their geographic location. Households were randomly selected and invited to participate in the study. Households with no adult present were excluded from the survey and were replaced by the neighbouring households. Individual signed consent was obtained from all adults or guardians of household member under 18 years of age. Samples were collected from all household members present aged over 6 months. The minimum sample size of 439 individuals per sub-district was met to ensure the antibody SCR of 0.0036 could be estimated with a precision level of ± 0.0013 [35]. Standard microscopy blood smears were collected as per routine national diagnostic standards. Filter paper bloodspots were collected on Whatman 3 M paper (Whatman, UK) as described by Corran et al. [36] and stored at − 20 °C until transferred to the Parasitology Laboratory at Department of Parasitology, UGM, Yogyakarta. Data on age, gender, education, occupation, long-lasting insecticide-treated bed net (LLIN) use, indoor residual spraying (IRS) in last 12 months and auxiliary temperature were recorded using a short questionnaire form, and household GPS coordinates were collected using handheld GPS.

### Laboratory methods

Giemsa-stained thick and thin malaria films reading was performed by trained laboratory technicians to identify active infections. For serological assays, the recombinant proteins *Plasmodium falciparum* MSP-1_19_, *P. falciparum* AMA-1, *Plasmodium vivax* MSP-1_19_ and *P. vivax* AMA-1 were used as antigens in indirect enzyme-linked immunosorbent assay (ELISA) as described in [9]. Briefly, antigens were coated on 96 well plates at the concentration of 0.5 µg/mL in coating buffer and incubated at 4 °C overnight. The plates were washed in phosphate buffered saline with tween (PBST) and blocked with 1% (w/v) skimmed milk solution for 3 h. After washing, samples were added in duplicate at a final dilution of 1:1000 to each plate using a pool of hyperimmune serum as a positive control and the plates were incubated overnight at 4 °C. The plates were washed and 50 µL of HRP-conjugated rabbit anti Human IgG (DAKO, #P0214) were added into each well and incubated for 3 h. After a further series of washes substrate solution (OPD, Sigma #P8287, in PBS) was added and the reaction was allowed to develop for 15–20 min before addition of stopping solution (2 M H_2_SO_4_). The optical density was read using ELISA reader at 450 nm. All serology was performed by trained laboratory technicians at the Department of Parasitology, UGM, Yogyakarta.

### Statistical analysis

All statistical analyses were conducted in Stata IC 15 (Stata Corp, College Station, TX, USA). Infants under 1 year of age were excluded from each dataset to remove any influence of maternally derived antibodies [10]. Raw OD measurements were averaged and normalized against the positive control curve on each plate. A cut-off for seropositivity was determined for each antigen by calculating the mean plus 3 standard deviation values of OD values from serum samples of 40 Javanese individuals who had no history of travel to malaria endemic areas in Indonesia. Cut-offs were generated separately for each antigen [13]. Individuals were categorized as seropositive for *P. falciparum* if their antibody responses were above the cut-off for PfAMA-1 and/or PfMSP-1_19_ and seropositive for *P. vivax* if their antibody responses were above the cut-off for PvAMA-1 and/or PvMSP-1_19_. Seroconversion rates were estimated by fitting a simple reversible catalytic model to seroprevalence data for each antibody [10]. Models with two SCRs allowing detection of changes in SCR were fitted and a likelihood test ratio was performed to decide the most appropriate model. Bivariate and multivariable analysis were performed to identify potential factors associated with *P. falciparum* (and *P. vivax*) exposure among study participants. Logistic regression models were performed to estimate odds ratios (ORs) of factors associated with being seropositive to *P. falciparum* or *P. vivax,* respectively. Adjusted odds ratios (aORs) were obtained using a multivariable model, including the following covariates: age, gender, seropositivity to *P. vivax*, education status, employment status, LLIN use, IRS in last 12 months, fever status, and altitude. Samples from participants aged under 18 years old were excluded from analysis of education and employment status. Statistically significant variables (p < 0.05) detected in bivariate analysis were included in a multivariable model. The final model was developed using the forward stepwise approach which compared multivariable models to the most significant bivariate model using p-values calculated from likelihood ratio tests. Scatter plots matrix and coefficient correlation analysis were done to assess potential cross-reactivity between *P. falciparum* and *P. vivax* antigens.

### Spatial analysis

The spatial software SaTScan (v.9.4.2) was used to detect clusters of higher than average age-adjusted antibody responses to PfAMA-1, PfMSP-1_19_, PvAMA-1 and PvMSP-1_19_. The Normal model was used to detect clusters of households with higher than average age-adjusted antibody responses to PfAMA-1, PfMSP-1_19_, PvAMA-1, PvMSP-1_19_ antibody responses. This method has been previously utilized in several studies investigating malaria transmission heterogeneity in low endemic setting [25, 37]. Antibody responses data were first log10 transformed and then adjusted for age. The residuals from linear regression (log titre regressed against age in years, performed in Stata IC 15) were used to determine whether antibody responses were higher or lower than expected for any given age assuming a homogeneous distribution of risk. Residuals less than zero represent individuals whose responses were lower than or average for their age group whilst residuals above zero represent individuals whose responses were higher than average. These data were then averaged per household and categorized, based equally around the median, as ‘lower than average’, ‘average’, ‘slightly higher than average’, ‘higher than average’, and ‘much higher than average’ to generate an antibody response heat map. The scan statistic was set to calculate non-overlapping, statistically significant (p < 0.05) clusters with a maximum set radius of 3 km and with minimum 2 observations detected in a cluster. Data generated from SatScan were then plotted using ArcGIS software (v10.5).

## Results

### Study population

General characteristics of the sampled population is presented in Table 1. There were 1624 samples collected in the surveys from 605 households. The average number of people sampled per household was 3 (SD: 1.64). The proportion of females sampled (61%) was slightly higher than males. The majority of the samples came from Sukajaya sub-district (63%), and the median age of participants was 22 years (IQR: 9–38). Educational attainment was high, with only 0.34% of adults ≥ 18 years old who had not completed primary education. More than half (57%) of the working-age population (≥ 18 years old) were unemployed. The population LLIN coverage was 60%, with 68% of those who owned nets reporting to have slept under it the night before. Only 15% of study households had received IRS in the previous 12 months. 9% of the population had fever with body temperature reading > 37.5 °C. Examination of microscopy slides found no malaria infections.

**Table 1:** Demographic characteristics and factors associated with *P. falciparum* transmission in Sabang, Indonesia, 2013

Variable (n = 1624)	Total	*P. falciparum* seropositive		Bivariate	Multivariable
	N (%)	n	% (95% CI)	OR (95% CI)	aOR (95% CI)
Age (years)					
< 15 years old	656 (40.39)	8	1.2 (0.6–2.4)	1	1
24 years old	270 (16.63)	19	7.0 (4.5–10.8)	6.13 (2.65–14.17)**	5.69 (2.43–13.37)**
25–40 years old	347 (21.37)	45	13.0 (9.8–16.9)	12.07 (5.63–25.88)**	12.05 (5.59–25.94)**
> 40 years old	351 (21.61)	40	11.4 (8.5–15.2)	10.4 2 (4.83–22.48)**	10.27 (4.74–22.27)**
Gender					
Female	984 (60.55)	71	7.2 (5.8–9.0)	1	
Male	641 (39.45)	41	6.4 (4.7–8.6)	0.88 (0.59–1.31)	
*P. vivax* seropositive					
No	1592 (98.03)	104	6.5 (5.4–7.9)	1	1
Yes	32 (1.97)	8	25.0 (12.9–42.9)	4.77 (2.09–10.88)**	3.47 (1.48–8.12)**
Residence					
Sukakarya	603 (37.13)	40	6.6 (4.9–8.9)	1	
Sukajaya	1021 (62.87)	72	7.1 (5.6–8.8)	1.07 (0.72–1.59)	
Education					
None	3 (0.34)	0	0	1	
Primary education	764 (86.33)	85	10.9 (8.9–13.2)	0.98 (0.53–1.83)	
Higher education	118 (13.33)	13	11.0 (6.5–18.1)	1	
Employment					
Unemployed	516 (57.33)	58	11.2 (8.8–14.3)	1	
Non-office-based job	215 (23.89)	27	12.6 (8.7–17.7)	1.13 (0.70–1.85)	
Office-based job	105 (11.67)	9	8.6 (4.5–15.7)	0.74 (0.35–1.55)	
Student	64 (7.11)	5	7.8 (3.3–17.6)	0.67 (0.26–1.74)	
LLIN use					
No	1098 (68.28)	63	5.7 (4.5–7.3)	1	1
Yes	510 (31.72)	48	9.4 (7.2–12.3)	1.71 (1.16–2.52)**	1.80 (1.20–2.72)**
IRS last 12 months					
No	1376 (84.83)	93	6.8 (5.6–8.2)	1	
Yes	246 (15.17)	19	7.7 (5.0–11.8)	1.15 (0.69–1.93)	
Fever					
No	1483 (91.26)	102	6.9 (5.7–8.3)	1	
Yes	142 (8.74)	10	7.0 (3.8–12.6)	1.03 (0.52–2.01)	
Altitude (m)					
< 120	716 (50.46)	50	7.0 (5.3–9.1)	1	
> 120	703 (49.54)	44	6.3 (4.7–8.3)	0.89 (0.99–1.00)	

* p value < 0.05, ** p value < 0.01. Individual level data: age, gender, education status, employment status and fever. Household level data: LLIN use, IRS in last 12 months and altitude

### Seroprevalence and associated factors

Seropositivity to *P. falciparum* antigens was higher than seropositivity to *P. vivax* antigens, with seroprevalence 6.89% (95% CI 5.76–8.24) and 1.97% (95% CI 1.39–2.77), respectively. Seroprevalence ranged from 1.2 to 11.4% for *P. falciparum* and 0.5 to 2.8% for *P. vivax* across age groups. Notably, there were no seropositive individuals aged under 5 years old identified for either *P. falciparum* or *P. vivax* (0/210). Seroprevalence to each antigen can be found in Additional file 1.

Multivariable analysis in Table 1 shows that age, seropositivity to *P. vivax* and use of LLINs were significantly associated with *P. falciparum* seropositivity, after controlling for other covariates. As would be expected, seroprevalence increased with age. Adults were more likely to be seropositive compared to children under 15 years old, with adjusted OR 5.69 (95% CI 2.43–13.37), 12.05 (95% CI 5.59–25.94) and 10.27 (95% CI 4.74–22.27) for age group 16–24, 25–40 and over 40 years old, respectively. Seropositivity to *P. falciparum* was also significantly associated with higher proportion of LLIN use, with adjusted OR 1.80 (95%: 1.20–2.72). In addition, people who were seropositive to any *P. vivax* antigen were 3 times more likely to be seropositive for *P. falciparum*, with adjusted OR 3.47, (95% CI 1.48–8.12). Other factors such as gender, residence, education, employment, IRS, fever and altitude were not significantly associated with *P. falciparum* seropositivity. Multivariable logistic regression revealed that there were no factors significantly associated with *P. vivax* seropositivity (Additional file 2).

### *Plasmodium falciparum* and *Plasmodium vivax* transmission intensity

**Fig. 2:**
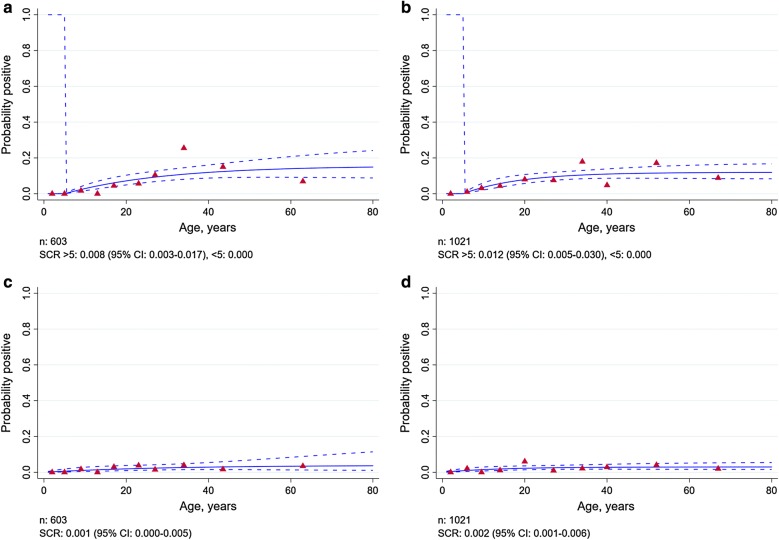
Age-seroprevalence plots for *P. falciparum* in Sukakarya (**a**), Sukajaya (**b**), for *P. vivax* in Sukakarya (**c**) and in Sukajaya (**d**), 2013. Solid lines represent the fitted probability for being seropositive to either MSP-1_19_ or AMA-1 antigen, dashed lines represent the 95% confidence interval of these fits and red triangles represent the observed proportion of seropositive per age decile. SCR value represent the average annual rate at which the population become seropositive to any of *P. falciparum* or *P. vivax* antigen

Figure 2 describes the SCR estimates for *P. falciparum* and *P. vivax* in Sukakarya and Sukajaya sub-districts, Sabang municipality, Indonesia in 2013. The SCR estimates suggested that there was a significant change in *P. falciparum* transmission in both Sukakarya and Sukajaya sub-districts, with no exposure seen in children under 5 years old. The data suggested that the *P. falciparum* transmission intensity in people aged over 5 years old was SCR 0.008 (95% CI 0.003–0.017) and SCR 0.012 (95% CI 0.005–0.030) in Sukakarya and Sukajaya, respectively. The SCR estimates for *P. vivax* (Fig. 2c, d) also suggested a very low level of transmission, SCR 0.001 (95% CI 0.000–0.005) and 0.002 (95% CI 0.001–0.006), respectively. There was no evidence for a difference in SCR between people aged under 5 and over 5 years old in either Sukakarya or Sukajaya. Overall, these model SCRs estimates suggested that the magnitude of transmission in population level was likely to be similarly very low for *P. falciparum* and *P vivax*.

### Heterogeneity of *P. falciparum and P. vivax*

**Fig. 3:**
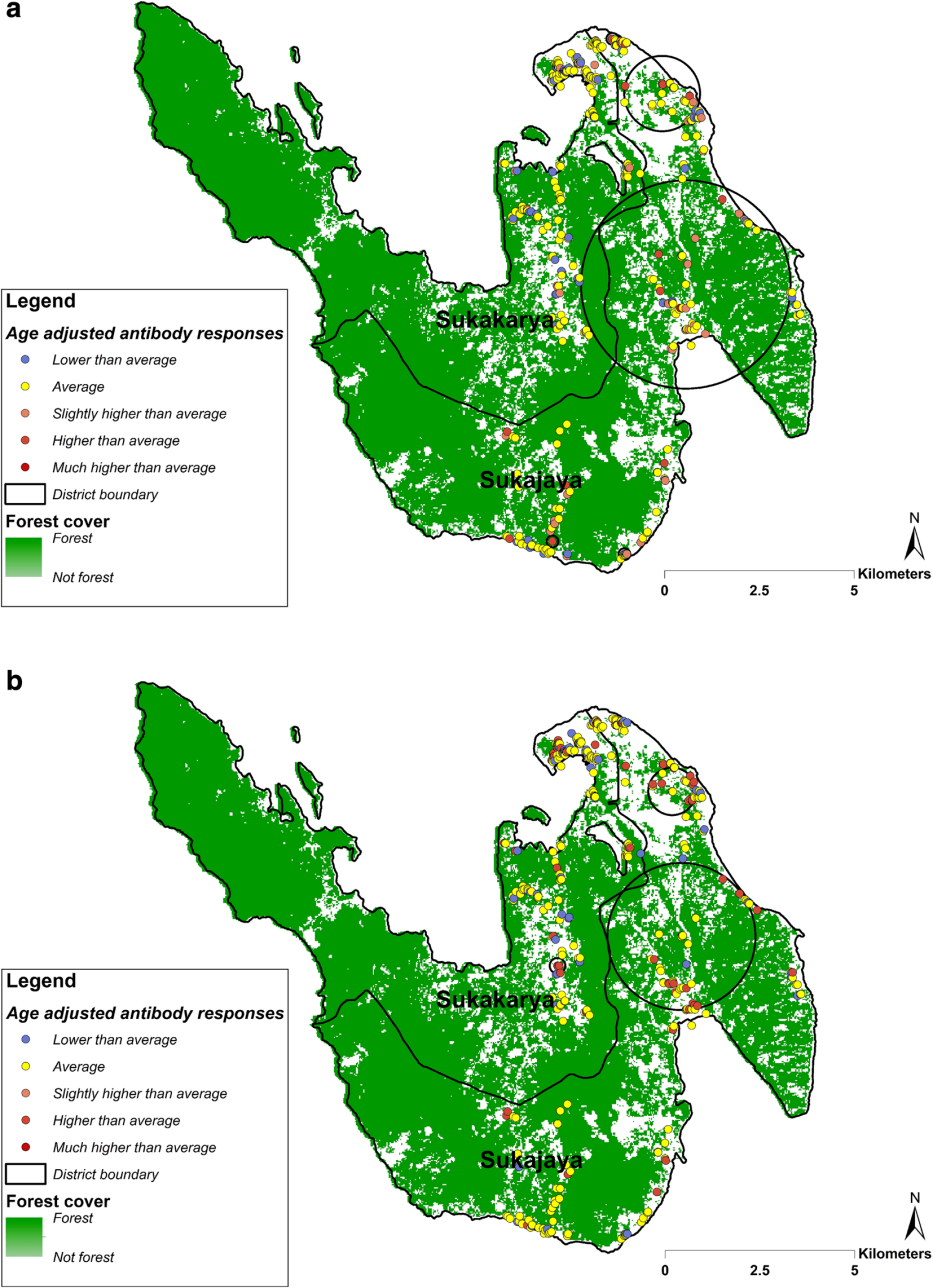
Spatial distribution of household-averaged, age-adjusted antibody responses to **a** PfAMA-1 and to **b** PfMSP-1_19_ in Sukakarya and Sukajaya sub-districts, Sabang, Indonesia. The resultants residual values were categorized as: ‘lower than average’ (− 4.326 to − 0.499), ‘average’ (− 0.500 to 0.500), ‘slightly higher than average’ (0.501 to 1.000), ‘higher than average’ (1.001 to 1.500) and much higher than average (1.501 to 2.842). Black circle indicates a cluster of significantly higher than expected antibody responses detected using SaTScan (p value < 0.05)

**Fig. 4:**
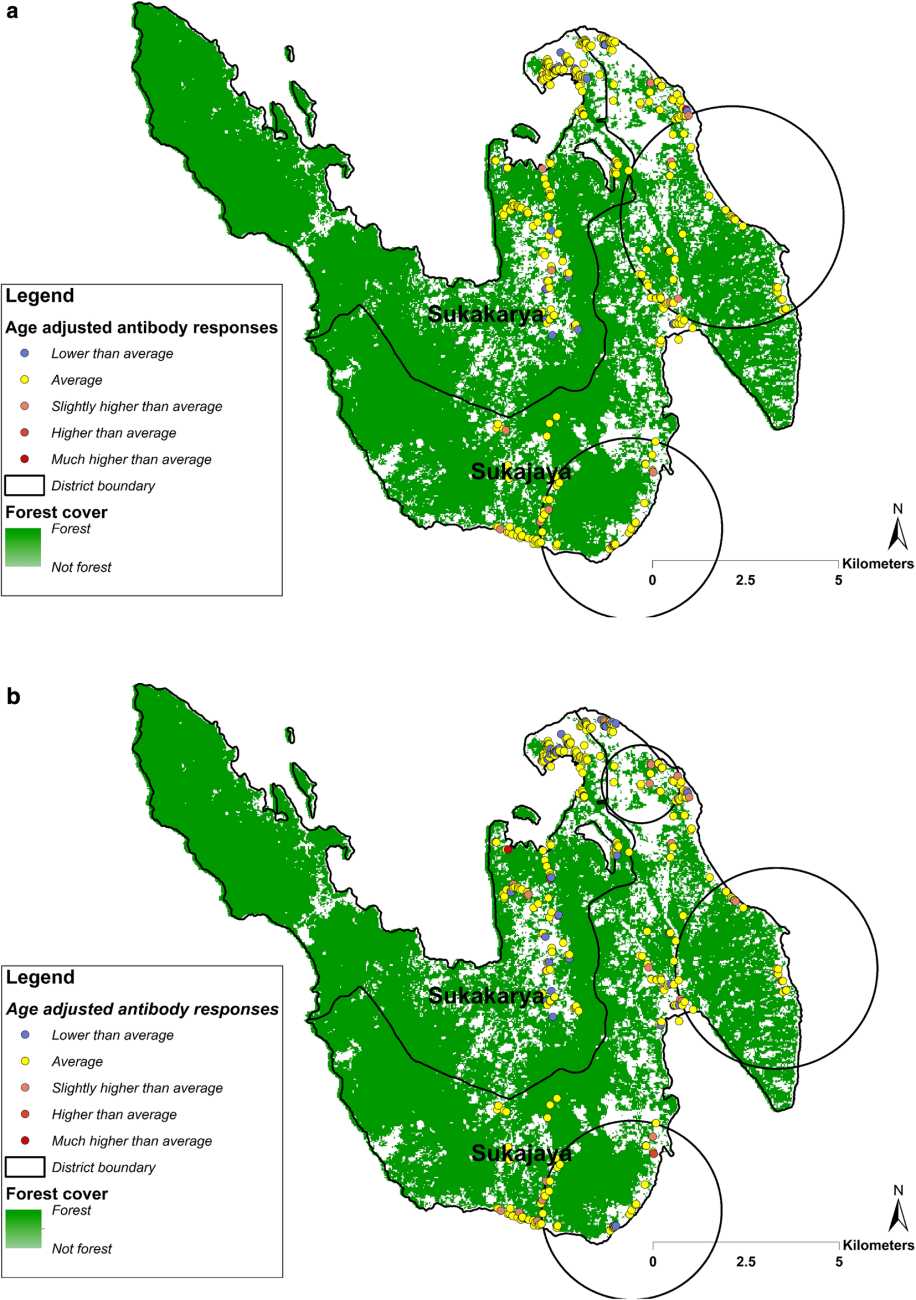
Spatial distribution of household-averaged, age-adjusted antibody responses to **a** PvAMA-1 and to **b** PvMSP-1_19_ in Sukakarya and Sukajaya sub-districts, Sabang, Indonesia. The resultants residual values were categorized as: ‘lower than average’ (− 4.933 to − 0.499), ‘average’ (− 0.500 to 0.500), ‘slightly higher than average’ (0.501 to 1.000), ‘higher than average’ (1.001 to 1.500) and much higher than average (1.501 to 2.117). Black circle indicates a cluster of significantly higher than expected antibody responses detected using SaTScan (p value < 0.05)

Spatial analysis of higher than average age-adjusted antibody responses identified 5 significant clusters for PfAMA-1 and 3 clusters for PfMSP-1_19._ All 5 of the PfAMA-1 clusters were seen in Sukajaya (Fig. 3a), whilst 2 of 3 PfMSP-1_19_ clusters seen in Sukajaya and spatially overlapped with the PfAMA-1 clusters (Fig. 3b). One additional PfMSP-1_19_ cluster was identified in Sukakarya. The analysis of age adjusted antibody responses to *P. vivax* antigens identified 2 clusters for PvAMA-1 and 3 clusters for PvMSP-1_19_ in Sukajaya (Fig. 4). The clusters identified for PvAMA-1 spatially overlapped the PvMSP-1_19_ clusters. All of these *P. vivax* clusters were seen in Sukajaya, whilst no clusters were identified in Sukakarya. Overall, the clusters identified for *P. falciparum* and *P. vivax* were seen in the same areas.

## Discussion

This study describes the analysis of community-based serological data to investigate malaria transmission dynamics in a low transmission setting, Sabang, Indonesia. The seroprevalence and SCR data represent exposure to infection and demonstrate that the population level of transmission intensity were similarly very low for both *P. falciparum* and *P. vivax.* The seroprevalence in children under 15 years old was negligible, 1.2% and 0.5% for *P. falciparum* and *P. vivax*, respectively. The spatial analysis of household-level data on antibody responses to any of the antigens tested describe the heterogeneity of both *P. falciparum* and *P. vivax* exposure in the study area. These results supported previous utilization of sero-epidemiological analysis in assessing population–level transmission intensity and differentiating between areas of different endemicity in Indonesia [30]. Moreover, multivariable analysis utilizing serological and epidemiological data collected through community-based survey identified that age, *P. vivax* seropositivity and LLIN use were significantly associated with *P. falciparum* seropositivity. These associations are likely related to historical exposure as *P. falciparum* seroprevalence was estimated to be low and parasite screening found no active infections detected by microscopy. Although sub-microscopic infections might present in the community, a previous study suggested that the proportion of sub-microscopic infections detected via PCR (polymerase chain reaction) was very low 0.07% (11/16,229) in the region [34].

The *P. falciparum* SCR estimates suggest that there was no exposure seen in children under 5 years old in both sub-districts in Sabang municipality. These results could represent a step change in *P. falciparum* transmission due to the successful impact of malaria control programme implemented in the study area, evidenced by lower antibody prevalence in children born after the intervention scale-up. This finding was supported by a previous study documenting a significant drop in malaria cases after the launch of the control program in 2004. Malaria cases in Sabang declined from 88 cases per 1000 population in 2004 to 1 per 1000 by 2010. The decline in malaria transmission in Sabang is likely related to an extensive IRS programme immediately following the tsunami in 2004, large scale LLIN distribution, and a change in malaria treatment policy to artemisinin-based combined therapy as first-line treatment for uncomplicated malaria [34]. Sabang was certified as a malaria-free region by the Indonesian Government as a result of successfully maintaining zero cases since the last locally transmitted case reported in 2011. Since then, the surveillance system detected 12 imported cases consisting of 6 *P. vivax*, *4 P. falciparum* and 2 mixed *P. vivax* and *P. falciparum* infections from 2011 to 2013, with no local transmission. However, the surveillance system detected 15 PCR confirmed *Plasmodium knowlesi* infections that classified as an outbreak in 2014 [38].

Consistent with the higher *P. falciparum* SCR estimates in people over 5 years old, multivariable analysis revealed that adults were more likely to be seropositive compared to children under 15 years old. This is likely the result of higher exposure by staying overnight in high-risk areas. A recent study revealed that the clusters of malaria (*P. knowlesi)* infections in Sabang was associated with people who had a history of staying overnight in the forest, without protection from mosquitoes, in an area where macaques are common [38]. Unfortunately, data on travel behaviour and occupation in these surveys were not recorded to enable testing of these hypotheses. Future research would need to include more detailed questions regarding travel behaviour, occupation and other essential risk factor data such as travel history to high-risk areas, night outdoor activities, sleeping in plantation or forest, housing, personal protection, etc. Several programme initiatives, for example a multi-country study on vector control tools to address outdoor transmission and project management quality improvement for national malaria program workforce carried out under the Asia Pacific Malaria Elimination Network would be beneficial for the malaria elimination effort in the region. In addition, the use of LLIN was almost two times higher in area where *P. falciparum* seroprevalence was higher. Consistent with previous report suggesting high coverage of LLINs (over 75%) in six malaria focal villages in Sabang, this finding suggests that people living in higher risk of exposure were aware of the importance of LLIN to prevent malaria transmission in those areas [34].

The estimated age-seroprevalence curves and SCR value suggested that age was not associated with *P. vivax* transmission in either sub-district in Sabang. *Plasmodium vivax* seroprevalence was very low (2.0%) and, therefore, the absence of any associations is likely due to the statistical limitations of the low number of seropositive samples. The other possible explanation is that *P. vivax* infections may induce lower antibody responses or shorter-lived responses which the current assay may miss. Work is ongoing to identify *P. vivax* antigens that elicit short-term responses for easy identification of very recent exposure [39, 40]. The need for testing more potential *P. vivax* antigens is supported by a previous study showing that the number of *P. vivax* cases tend to be higher than the number of *P. falciparum* cases in Sabang [34].

The spatial analysis of age-adjusted antibody responses to either antigen (AMA-1 or MSP-1_19_) identified significant clusters of higher exposure (hotspots) for both *P. falciparum* and *P. vivax* exposure across the study areas. Although multivariable risk factors analysis found there was no significant association between residence and higher seroprevalence to *P. falciparum* and *P. vivax*, the spatial analysis suggested that the risk of malaria transmission in the study setting is heterogeneous with people experiencing higher exposure in Sukajaya sub-district. The spatial analysis also suggest that the clusters identified for *P. falciparum* and *P. vivax* were seen in the same areas. Being able to characterize the micro-epidemiology of malaria exposure could assist malaria control programme to better allocate resources and target the intervention to achieve their goal of elimination. Targeting hotspots could be a highly efficient way to reduce malaria transmission at all levels of transmission intensity [41]. Although this study identified potential high-risk areas using historical data collected in 2013, being able to identify areas which had the most recent exposure is useful for malaria surveillance. A recent study suggested that one of two clusters of *P. knowlesi* infections in Sabang were identified in similar high-risk areas identified in this study [38]. As suggested in the latest WHO malaria surveillance manual [11], maintaining surveillance activities in the most receptive areas could be useful to prevent potential reintroduction or resurgence of the disease in the future. Therefore, utilizing antibody responses data to identify recent or historical hotspots of transmission could be a powerful alternative approach where gaining direct evidence of an increased exposure to infectious mosquito bites is no longer ideal in low transmission settings.

Finally, people who were seropositive to any *P. vixax* antigen were 3 times more likely to be *P. falciparum* seropositive, after controlling for age, gender, residence, employment, education, IRS, fever status, and altitude. In addition, clusters of high antibody responses suggest that *P. falciparum* and *P. vivax* receptive areas were seen in the same areas. As there was no cross-reactivity evident from the serological data (Additional file 3), these findings could suggest that people were historically exposed to both infections, potentially due to the presence of efficient vectors in those identified areas.

Findings in this study are based on community-based samples and data collected during the malaria transmission season. Although this study describes the potential use of serological data analysis in estimating malaria transmission intensity, heterogeneity and factors associated to disease exposure, the results generated would need to be carefully interpreted. Previous studies suggested that malaria transmission in other areas of Indonesia was affected by seasonality [30, 34, 42–44] and behavioural factors such as farm or forest-related night outdoor activity (e.g. sleeping in forest gardens) [45, 46] and domestic travel to higher endemic areas [47]. However, due to limited data collected, our study could not examine the effect of behavioural factors such as forest-related activities or recent travel history to high-risk areas outside Sabang. Therefore, future studies measuring population level antibody responses coupled with collecting more data that could describe behavioural factors associated to higher risk of exposure would be more epidemiologically informative to assist malaria surveillance and control programme to achieve elimination in the region.

## Conclusion

In conclusion, these data add to the body of evidence that sero-epidemiological analysis of community-based surveys are an important additional tool to investigate malaria transmission dynamics in area aiming for elimination in Indonesia. Recent identification of alternative antigens associated with short-lived antibody responses suggests a potentially key indicator of very recent exposure which would be a very important information for public health surveillance [48]. The addition of a novel panel of *P. knowlesi* antigens [49] would enhance understanding of malaria transmission dynamics as recent studies reported that although laboratory identification of *P. knowlesi* in Indonesia is challenging [50], surprisingly, there were two clusters of *P. knowlesi* cases detected in Sabang after the municipality successfully eliminated *P. falciparum* and *P. vivax* cases [38]. Moreover, another recent study also reported there was a considerable proportion of *P. knowlesi* infection in another western part of Indonesia, in North Sumatera province [51]. Exploratory work employing techniques such as multiplex fluorescent magnetic bead-based serological assay to investigate and validate a panel of potential antigens for these applications is underway [40, 52]. The development and validation of a standardized serological sample and data collection methods utilizing existing public health surveillance system, for example as described in [53] will also facilitate the optimization of serological surveillance in understanding transmission dynamics to support malaria control programme in achieving elimination.

## Additional files

**Additional file 1.** Seroprevalence for each antigen studied.

**Additional file 2.** Demographic characteristics and factors associated with *P. vivax* transmission in Sabang, Indonesia, 2013.

**Additional file 3.** Scatter plots matrix of antibody responses (optical density) to *P. falciparum* and to *P. vivax* antigens tested in the study describing the absence of cross-reactivity between the *P. falciparum* and *P. vivax* antigens.

## Data Availability

The datasets used and analysed during this study are not publicly available due to the inclusion of identifying information on individuals but are available from the corresponding author on reasonable request and approval from relevant ethics committees.

## Abbreviations

CI: confidence interval
EIR: entomological inoculation rate
IRS: indoor residual spraying
LLIN: long-lasting insecticide-treated bed nets
PfAMA-1_19_: *P. falciparum* apical membrane antigen 1
PfMSP-1: *P. falciparum* merozoite surface protein 1
PvAMA-1_19_: *P. vivax* apical membrane antigen 1
PvMSP-1: *P. vivax* merozoite surface protein 1
SCR: seroconversion rates
WHO: World Health Organization
