# Supplementary material for: Analysis of serological data to investigate heterogeneity of malaria transmission: a community-based cross-sectional study in an area conducting elimination in Indonesia
Source: Malar J. 2019 Jul 8;18:227. doi: 10.1186/s12936-019-2866-z (PMC6615161; doi:10.1186/s12936-019-2866-z)
Supplement: Supplementary file 1 — Additional file 1. Seroprevalence for each antigen studied. [file 12936_2019_2866_MOESM1_ESM.docx]

**Additional file 1.** Seroprevalence for each antigen studied

| **Antigen** | **N** | **Number**  **positive** | **% (95% CI)** |
| --- | --- | --- | --- |
| PfAMA-1 | 1615 | 85 | 5.3 (4.3-6.5) |
| PfMSP-1_19_ | 1624 | 58 | 3.6 (2.8-4.6) |
| PvAMA-1 | 1624 | 19 | 1.2 (0.8-1.8) |
| PvMSP-1_19_ | 1624 | 17 | 1.2 (0.7-1.7) |

**Additional file 2.** Demographic characteristics and factors associated with *P. vivax* transmission in Sabang, Indonesia, 2013

| **Variable (n = 1624)** | **Total** | ***P. vivax* seropositive** | | **Bivariate** | ***p*** |
| --- | --- | --- | --- | --- | --- |
|  | **N (%)** | **n** | **% (95% CI)** | **OR (95% CI)** |  |
| Age (years)  <15 years old   - 1. years old   25-40 years old  >40 years old | 656 (40.39)  270 (16.63)  347 (21.37)  351 (21.61) | 3  11  8  10 | 0.5 (0.1-1.4)  4.1 (2.3-7.2)  2.3 (1.2-4.5)  2.8 (1.5-5.2) | 1  9.24 (2.56-33.41)  5.14 (1.35-19.49)  6.38 2 (1.75-23.35) | 0.001  0.016  0.005 |
| Gender  Female  Male | 984 (60.55)  641 (39.45) | 23  9 | 2.3 (1.6-3.5)  1.4 (0.7-2.7) | 1  0.59 (0.27-1.29) | 0.190 |
| Residence  Sukakarya  Sukajaya | 603 (37.13)  1021 (62.87) | 11  21 | 1.8 (1.0-3.3)  2.1 (1.3-3.1) | 1  1.13 (0.54-2.36) | 0.745 |
| Education  None  Primary education  Higher education | 3 (0.34)  764 (86.33)  118 (13.33) | 1  27  3 | 0.4 (0.1-2.6)  2.2 (1.5-3.2)  2.5 (0.8-7.5) | 1  6.14 (0.83-45.38)  6.97 (0.72-67.75) | 0.075  0.094 |
| Employment  Unemployed  Non-office-based job  Office-based job  Student | 516 (57.33)  215 (23.89)  105 (11.67)  64 (7.11) | 20  4  2  6 | 2.5 (1.6-3.8)  1.8 (0.7-4.8)  1.9 (0.5-7.1)  1.2 (0.6-2.7) | 1  0.73 (0.25-2.16)  0.73 (0.17-3.19)  0.48 (0.19-1.22) | 0.572  0.681  0.123 |
| LLIN use  No  Yes | 1098 (68.28)  510 (31.72) | 19  13 | 1.7 (1.1-2.7)  2.5 (1.5-4.3) | 1  1.48 (0.73-3.03) | 0.278 |
| IRS last 12 months  No  Yes | 1376 (84.83)  246 (15.17) | 25  7 | 1.8 (1.2-2.7)  2.8 (1.4-5.9) | 1  1.58 (0.68-3.70) | 0.290 |
| Fever  No  Yes | 1483 (91.26)  142 (8.74) | 31  1 | 2.1 (1.5-3.0)  0.7 (0.1-4.8) | 1  0.33 (0.45-2.45) | 0.280 |
| Altitude (meter)  < 120  > 120 | 716 (50.46)  703 (49.54) | 13  10 | 1.8 (1.1-3.1)  1.4 (0.8-2.6) | 1  0.78 (0.34-1.79) | 0.559 |

Individual level data: age, gender, education status, employment status and fever. Household level data: LLIN use, IRS in last 12 months and altitude

**
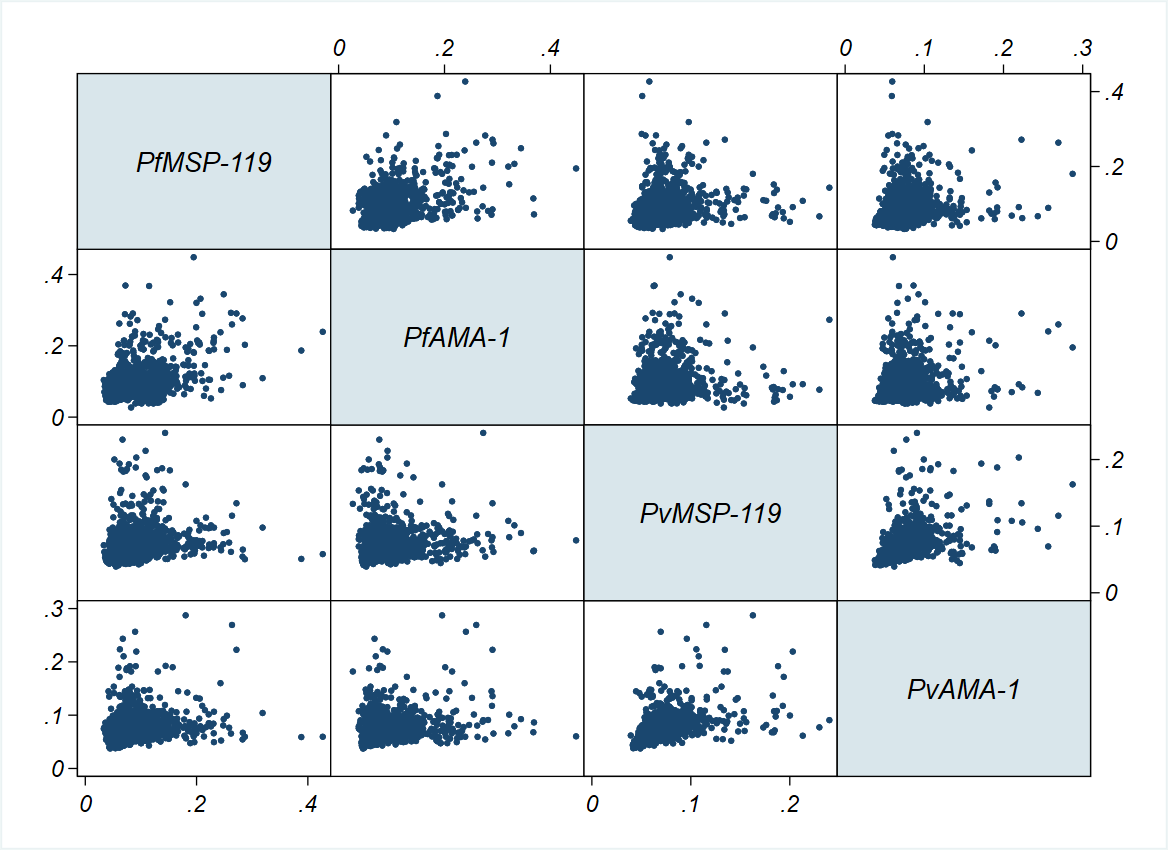
Additional file 3.** Scatter plots matrix of antibody responses (optical density) to *P. falciparum* and to *P. vivax* antigens tested in the study describing the absence of cross-reactivity between the *P. falciparum* and *P. vivax* antigens
