# Supplementary material for: Analysis of serological data to investigate heterogeneity of malaria transmission: a community-based cross-sectional study in an area conducting elimination in Indonesia
Source: Malar J. 2019 Jul 8;18:227. doi: 10.1186/s12936-019-2866-z (PMC6615161; doi:10.1186/s12936-019-2866-z)
Supplement: Supplementary file 3 — Additional file 3. Scatter plots matrix of antibody responses (optical density) to P. falciparum and to P. vivax antigens tested in the study describing the absence of cross-reactivity between the P. falciparum and P. vivax antigens. [file 12936_2019_2866_MOESM3_ESM.docx]

**
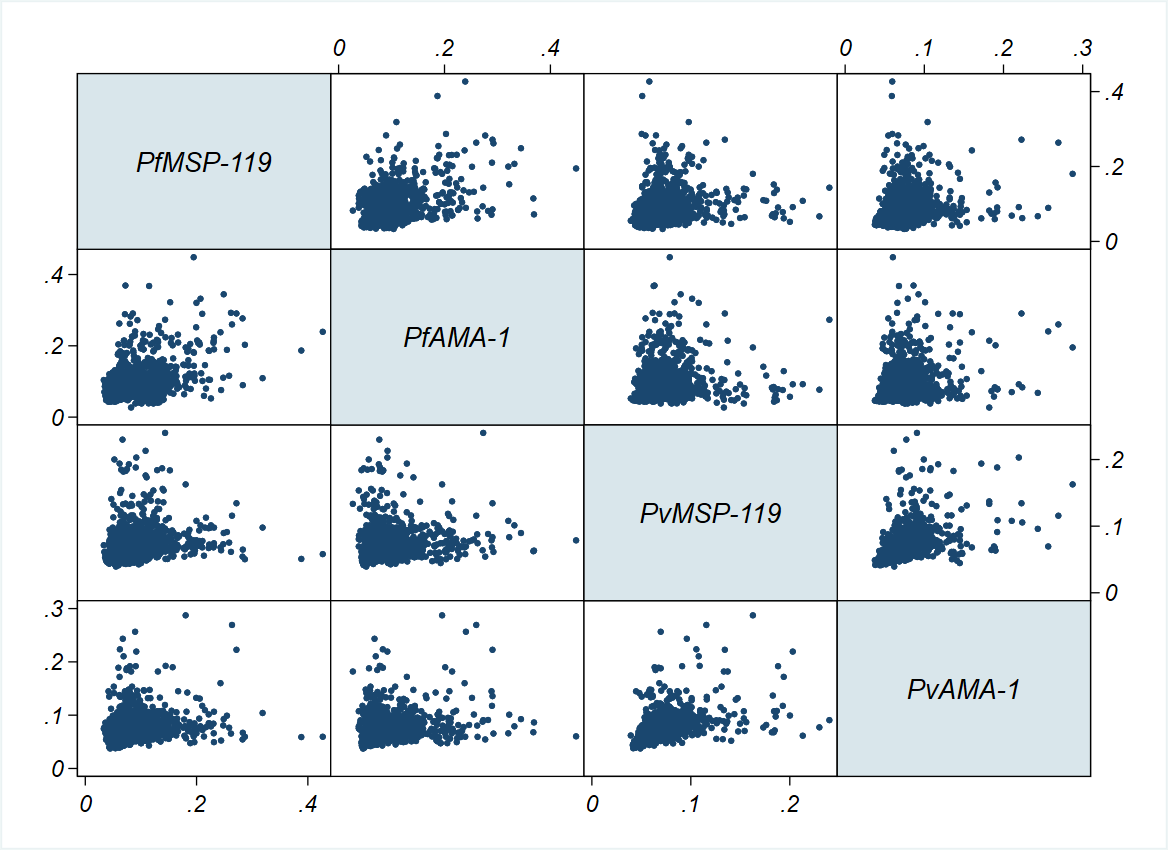
Additional file 3.** Scatter plots matrix of antibody responses (optical density) to *P. falciparum* and to *P. vivax* antigens tested in the study describing the absence of cross-reactivity between the *P. falciparum* and *P. vivax* antigens
